# Optimized detection of bacteria in bloodstream infections

**DOI:** 10.1371/journal.pone.0219086

**Published:** 2019-06-26

**Authors:** Yajing Song, Peter Gyarmati

**Affiliations:** University of Illinois College of Medicine, Peoria, IL, United States of America; Academic Medical Centre, NETHERLANDS

## Abstract

Bloodstream infection (BSI) is a life-threatening condition characterized by the presence of pathogens in the blood. It is associated with increased morbidity and mortality, and has to be treated promptly as mortality increases with every hour of delayed treatment. Therefore, rapid and sensitive diagnosis of BSI is essential. The routine diagnostic method for BSI is blood culture, which can only detect culturable pathogens and takes several days to obtain results. The 16S rRNA gene is present in all bacteria and is commonly used as a target for universal bacterial detection in rapid molecular assays such as PCR. However, molecular detection of the 16S gene is hampered by the large amount of human DNA found in blood samples, making diagnostic results aspecific and less sensitive. We have optimized the selection of PCR primers targeting the 16S rRNA gene to avoid cross-reaction with human DNA background. The developed method increases specificity and sensitivity for pathogen diagnosis, and provides rapid and accurate pathogen detection for rare bacterial DNA in the presence of abundant host DNA.

## Introduction

Bloodstream infection (BSI) is a severe medical condition associated with increased morbidity and mortality worldwide, with and incidence of 80–200 per 100,000 annually [1]. The routine detection method of BSI is blood culture, and the most commonly detected bacterial strains are *Escherichia coli*, *Staphylococcus aureus* and *Streptococcus pneumoniae* [1]. Blood culture can only detect culturable pathogens, and it takes several days to perform. Molecular methods can also be used for diagnosing BSI: they can be executed in a few hours, and can detect a wide variety of bacteria by targeting multiple strains using specific probes or universal marker genes [2].

The 16S rRNA gene is a ubiquitous gene present in all bacteria, and therefore is often used for universal bacterial detection in BSI. However, there is a large amount of host DNA present in blood samples, which hampers the sensitivity and specificity of molecular BSI diagnosis [3, 4], due to the similarity between bacterial and human genomes [5, 6]. The goal of the current study was to select primers which efficiently amplify the 16S rRNA gene, but do not cross-react with the human genome.

Current approaches to avoid or reduce human DNA background in 16S detection of blood samples include the use of less PCR cycles what may result in reduced sensitivity, or enzymatic approaches, which may increase workload and cost [3, 4]. Our study indicates that a significant improvement in the sensitivity and specificity of bacterial DNA detection in BSI is achievable by avoiding cross-reactivity between PCR primers targeting the 16S rRNA gene and the host background DNA.

## Materials and methods

### Identification of 16S rRNA primers with no similarity to the human genome

All available primer sequences (n = 113) targeting the conserved segments of the 16S rRNA gene have been downloaded from ProbeBase (S1 Table, [7]). Similarity search to the human genome and its transcripts has been performed using BLAST [8]. In addition, sequences have also been mapped to the human genome (GRCh v38) using the CLC Genomic Workbench. Primer pairs were selected for further testing based on 1) their melting temperatures (≤ 10 C^o^ difference between forward and reverse primers) and 2) their sizes as our aim was to identify primer pairs for high throughput sequencing (≤550 base pair) and Sanger sequencing (≥700 base pair). Melting temperatures of primers were determined using OligoCalc [9].

### Cross-reactivity of primers with human genome

The specificity of the selected primers were tested in a PCR reaction with *E*. *coli* and human genome (Sigma) as templates. PCR conditions were the following: 200 nM primers, 1x Kapa PCR mix (Kapa Biosystems), 3 μl template in a 20 μl reaction. Tubes were incubated in a thermocycler at 95 ^o^C for 3 min, then cycled at 95 ^o^C for 30 sec, 50 ^o^C for 30 sec, and 72 ^o^C for 30 sec for 25 times.

Sensitivity of the primer pairs was tested with a mixture of human and *E*. *coli* genomes, by modeling conditions of BSI. Standard amount of human genome (11 ng/μl) and decimal dilutions of *E*. *coli* (55 ng/μl to 55 fg/μl) were used as templates. DNA extracted from 100 μl sterile human serum (Sigma) was also used to determine cross-reactivity of primers with human background DNA. The 341F and 803R primers were added as reference as they are widely used for sequencing studies [10, 11]. Tubes were incubated in a thermocycler at 95 ^o^C for 3 min, then cycled at 95 ^o^C for 30 sec, 50 ^o^C for 30 sec, and 72 ^o^C for 30 sec for 45 times. Signal intensities were measured using the myImageAnalysis software (Fisher Scientific). Positive signals were defined as ≥5% increase compared to the background signal.

### Sequencing

Two-hundred μl PCR amplicons were used for Sanger sequencing to identify the amplified products using an AB 3730xl instrument. Sequences were analyzed using the BioEdit 7.2.5 software [12]. The Sanger sequencing data has been uploaded to GenBank under accession numbers MK674079-MK674083 and MK672919-MK672927.

## Results

### Sequence similarity of primers with the human genome

One hundred-thirteen primers have been analyzed, and 7 primers (6.2%) were identified with 0% similarity (64F, 363F, 520F, 530F, 806R, 1027R, 1100R, Table 1.) to the human genome and its transcripts.

**Table 1:** Sequences of primers used in this study.

	Sequence 5´-3´
363F	CAA TGG RSG VRA SYC TGA HS
64F	BGY CTW ANR CAT GCA AGT YG
520F	AYT GGG YDT AAA GNG
530F	GTG CCA GCM GCN GCG G
806R	GGA CTA CHV GGG TAT CTA AT
1027R	CGA CRR CCA TGC ANC ACC T
1100R	GGG TTN CGN TCG TTG

### Cross-reactivity of primers with the human genome

Blood samples from BSI contain a large amount of human DNA background what can result in false positive (cross-reactivity with the human genome) or false negative (due to reduced sensitivity) detection of bacteria [3, 4]. We investigated the specificity of the selected primer pairs using *E*. *coli* and human genomic DNA.

**Fig 1:**
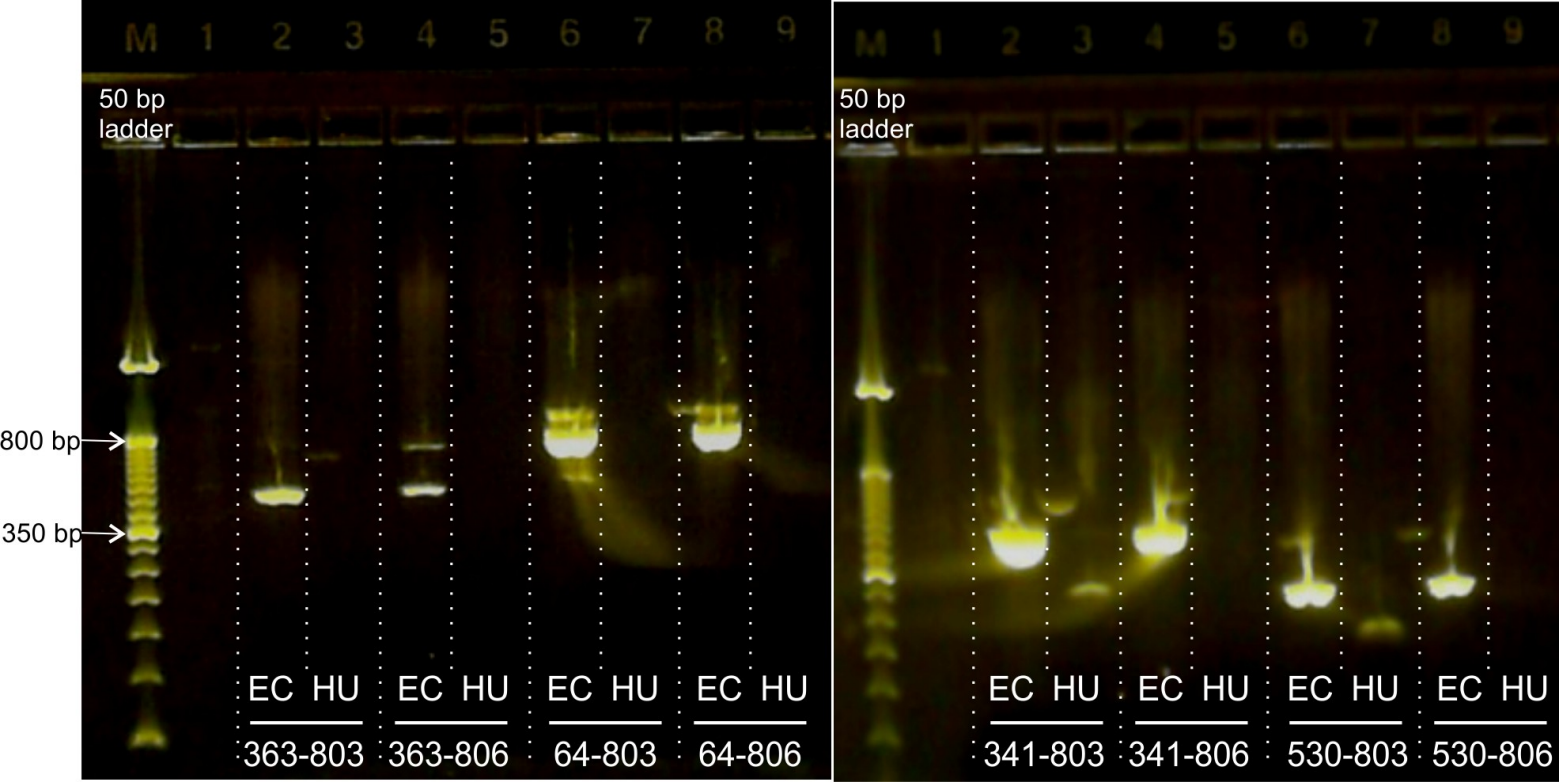
Gel electrophoresis on a 2% agarose gel shows amplification of *E*. *coli* (EC) and human genomic DNA (HU) with selected primer pairs. The 341–803 and 530–803 primer pairs resulted in misamplifications with human genomic DNA, while the 363–806 primers produced secondary PCR products. All other primer pairs resulted in specific amplification of the 16S rRNA gene. The concentrations of the samples were: 55 ng/μl for EC (165 ng total) and 11 ng/μl (33 ng total) for HU.

The reference primer 341F resulted in false amplification with all reverse primers, similarly to the 1027R primer, as it also generated false amplification in all combinations (Table 2, Fig 1). Apart from their size, the amplicons were also identified by Sanger sequencing, and their origins (human or bacterial) were confirmed in all cases. The following combinations specifically amplified *E*. *coli* but not the human genome: 64–803, 64–806, 363–803, 363–806, 530–806 (Table 2, Fig 1). The experiment was repeated with *Staphylococcus aureus* and *Campylobacter jejuni* genomic DNA as well with identical results.

**Table 2:** The table indicates whether different combinations of 16S primers resulted in an amplification when reacting with bacterial or human genome (+: positive reaction with *E*. *coli*/human genome,; -: no amplification with *E*. *coli*/human genome, green = forward, blue = reverse primer).

Forward (green)/Reverse (blue)	803	806	1027
64	+/-	+/-	+/+
341	+/+	+/+	+/+
363	+/-	+/-	+/+
530	+/+	+/-	+/+

### Sensitivity of PCRs with different primer pairs with human background DNA

**Fig 2:**
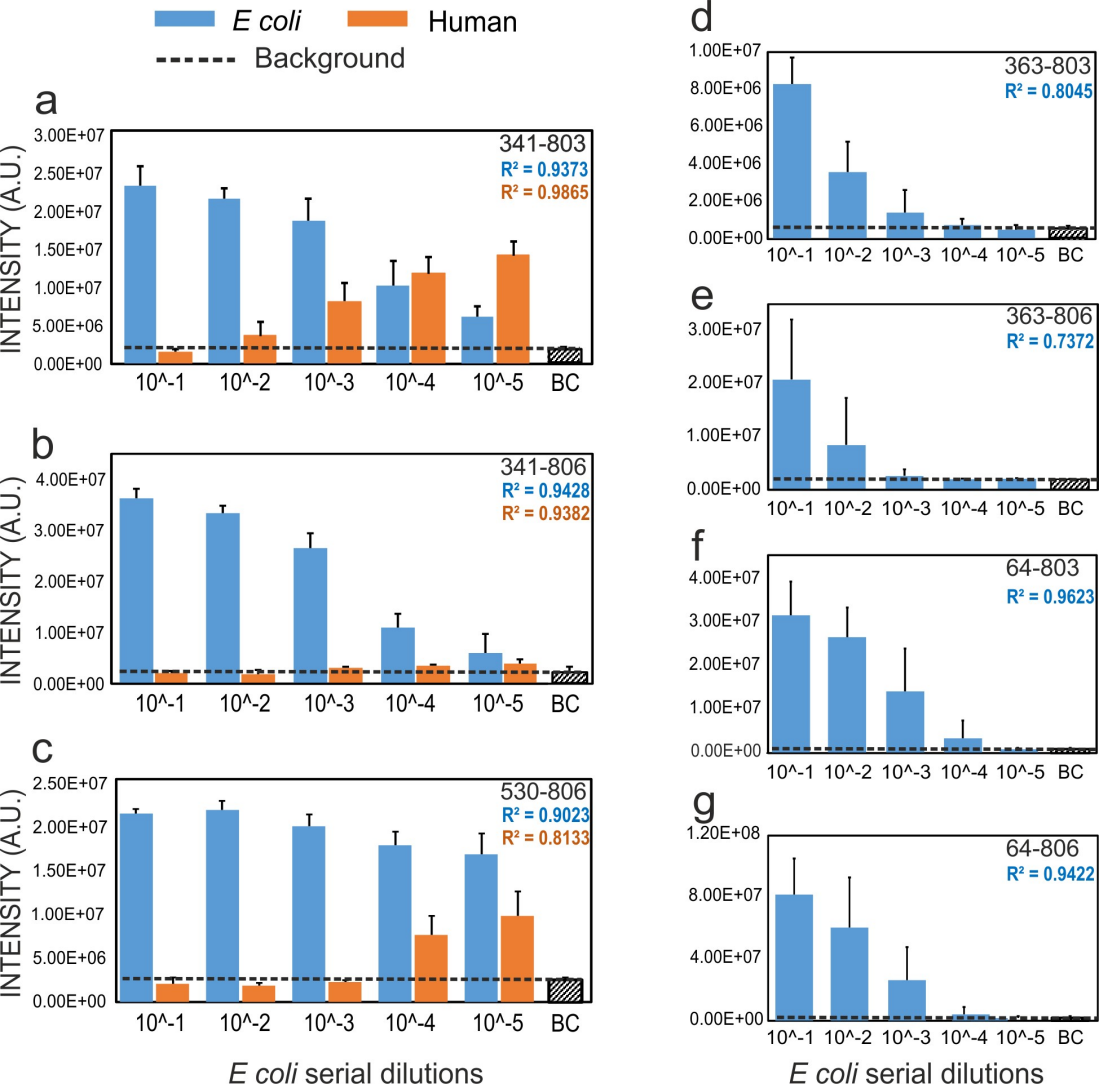
**Bars indicate signal intensities for human genome (red) in the presence of serial dilutions of *E*. *coli* (10**^**−1**^**–10**^**−5**^**, blue).** BC = background control. Primer pairs are shown in the top right corners. R^2^ values indicate linear regression.

Samples from BSI always contain a large amount of human DNA, which can result in misamplification, false positive results and decreased sensitivity of PCR systems [3, 4]. In order to model BSI, human DNA (in a standard amount) was mixed with serial dilutions of *E*. *coli* genomic DNA. Amplicons were subjected to Sanger sequencing and identified by BLAST. Sensitivity was calculated based on the resulted *E*. *coli* amplicons. Only the reference primer pair and those primers which did not cross-react with the human genome were tested. Triplicate trials resulted in misamplifications from 341–803, 341–806 and 530–806 (Fig 2, S1 Fig). Highest sensitivity for *E*.*coli* detection was achieved from 363–803, 64–803 and 64–806 (lowest concentration detected: 10^−4^). Lowest sensitivity was obtained from 341–803 (10^−2^ resulted in misamplification).

Serum samples resulted in amplification products from primer pairs 341–803, 341–806 and 530–806. Primer pairs 64–803, 64–806, 363–803 and 363–806 did not result in amplification with serum sample.

## Discussion

Rapid, sensitive and specific detection of pathogens is crucial in BSI cases. Antimicrobial treatment has to be started within hours after symptoms of BSI develops, because delay in antimicrobial treatment is associated with increased mortality in BSI [13]. Accurate identification of pathogens is important as false positive results would promote antibiotic misuse, generation of antimicrobial resistance and dysbiosis of the indigenous microbiota [14].

The routine BSI diagnostic method (blood culture) has limited detection range, requires extensive instrumentation and takes days to obtain results. Rapid molecular methods are available for BSI diagnosis, and can target universal bacterial marker genes, such as the 16S rRNA gene to allow the detection of unculturable pathogens [2].

However, contrary to most metagenomics samples (e.g., feces), samples such as blood contain a large amount of host DNA and low levels of bacterial DNA, which can decrease the specificity of molecular diagnostic systems due to cross-reactivity [3, 4]. Sensitivity is also crucial in BSI diagnosis, as pathogen concentration can be as low as 1 CFU/ml [15], and cross-reaction of primers results in less sensitive detection [3]. To address this problem, we optimized a bacterial detection system based on the amplification of the 16S rRNA gene, which show improved specificity (Fig 1) and sensitivity (Fig 2) by avoiding cross-reactivity with human genome.

Based on this work, using primer pairs such as the 363–803 or 363–806 for high-throughput sequencing systems and 64–803 or 64–806 for traditional Sanger sequencing provides increased sensitivity and specificity without amplifying human DNA (Fig 2).

In cases where the sizes of amplicons of human and bacterial samples are different (e.g., the 341–803 primer), size selection may be applicable to eliminate or decrease human background (Fig 1, 341–803 or 530–803 primer pairs), but it is a laborious process, with risk of PCR contamination. Our results show that amplicons with optimized 16S rRNA primers can be used without further size selection as they do not result in misamplification.

In summary, this work provides a sensitive and specific amplification of the 16S rRNA gene with appropriate selection of primers for BSI or other samples where a significant amount of human background DNA is present. These results can be utilized for molecular diagnosis of BSI, including high-throughput sequencing and PCR-based approaches.

## Supporting information

S1 Fig Sensitivity of 16S rRNA primer pairs. Gel images from triplicate trials show standard amount of human genomic DNA spiked in with *E*. *coli* dilutions (10^−1^–10^−5^) with different 16S primer pairs. White rectangles indicate 16S-specific amplicons. M = marker.
(TIF)

S1 Table Primer sequences and their melting temperatures used in this study. (PDF)
