# Supplementary material for: Optimized detection of bacteria in bloodstream infections
Source: PLoS One. 2019 Jun 26;14(6):e0219086. doi: 10.1371/journal.pone.0219086 (PMC6594679; doi:10.1371/journal.pone.0219086)
Supplement: S1 Table — (PDF) [file pone.0219086.s002.pdf]

Probebase Name	Sequence 5´-3´	Tm (Oligo Calc)
806R	GGA CTA CHV GGG TAT CTA AT	47.7-51.8
1027R	CGA CRR CCA TGC ANC ACC T	51.1-57.6
1100R	GGG TTN CGN TCG TTG	41.9-47.4
520F	AYT GGG YDT AAA GNG	33.7-44.7
530F	GTG CCA GCM GCN GCG G	53.6-58.8
64F	BGY CTW ANR CAT GCA AGT YG	45.6-55.9
363F	CAA TGG RSG VRA SYC TGA HS	49.7-60
Bakt 805R	GAC TAC HVG GGT ATC TAA TCC	50.5-54.4
U751F	CCG ACG GTG AGR GRY GAA	50.3-57.2
986F	CNA CGC GAA GAA CCT TAN C	48.9-53.2
6F	ATT CYG GTT GAT CCY GSC RG	51.8-57.9
P1425	WAG GAG GTR ATC CAD CC	44.6-49.5
802R	TAC NVG GGT ATC TAA TCC	43.5-48
P609D	GGM TTA GAT ACC CBD GTA	43.5-50.3
917Fw	GAA TTG ACG GGG RCC CGC A	55.4-57.6
Bac967Fd	ATA CGC GAR GAA CCT TAC C	48.9-51.1
803R	CTA CCR GGG TAT CTA ATC C	48.9-51.1
Bac1046Rd	CGA CGA CCA TGC ANC ACC T	53.2-55.4
907R	CCG TCA ATT CMT TTG AGT TT	45.6-47.7
68F	TNA NAC ATG CAA GTC GRR CG	47.7-55.9
P609R	TAC HVG GGT ATC TAA KCC	43.5-50.3
1061Rv	TCA CGR CAC GAG CTG ACG AC	55.9-57.9
1099F	GYA ACG AGC GCA ACC C	48.5-51.1
1114R	GGG TTG CGC TCG TTR C	48.5-51.1
Ab789F	TAG ATA CCC SSG TAG TCC	50.3
Bact531R	CTN YGT MTT ACC GCG GCT GC	53.8-60
1061R	CRR CAC GAG CTG ACG AC	49.5-54.3
U341F	CCT ACG GGR SGC AGC AG	54.3-56.7
Uni522R	GWA TTA CCG CGG CKG CTG	52.6-54.9
Bac1046Rb	CGA CAA CCA TGC ANC ACC T	51.1-53.2
Bac967Fe	CTA ACC GAN GAA CCT YAC C	48.9-53.2
Uni1392R	ACG GGC GGT GTG TRC	47.4-50.1
1050R	ACG ACA GCC ATG CAN C	45.9-48.5
1407R	GAC GGG CGG TGT GTR C	51.1-53.6
970F	CGC GAA GAA CCT TAC C	45.9
U1053F	GCA TGG CYG YCG TCA G	48.5-53.6
U1053R	CTG ACG RCR GCC ATG C	48.5-53.6
Bact806R	GGA CTA CCA GGG TAT CTA ATC CTG TT	58
908R	CGT CAA TTC MTT TGA GTT	41.2-43.5
536R	CAG CMG CCG CGG TAA TWC	52.6-54.9
909F	ACT CAA AKG AAT WGA CGG	43.5-45.8

U519F	CAG CMG CCG CGG TAA TWC	52.6-54.9
9bF	GRG TTT GAT CCT GGC TCA G	51.1-53.2
U779F	GCT AAS SGG ATT AGA TAC CC	51.8
39F	TGG CTC AGR WYG AAC GCT RG	51.8-57.9
41F	GCT CAG ATT GAA CGC TGG CG	55.9
U1517R	ACG GCT ACC TTG TTA CGA CTT	52.4
R357	CTG CTG CCT YCC GTA	44.7-47.4
917F	GAA TTG ACG GGG RCC C	48.5-51.1
527F	ACC GCG GCC KGC TGG C	56.2-58.8
1046R	CGA CAG CCA TGC ANC ACC T	53.2-55.4
Bac927R	ACC GCT TGT GCG GGC CC	56.7
Bac967Fc	CAA CGC GCA GAA CCT TAC C	53.2
GM12R	CGT CAT CCM CAC CTT CCT C	53.2-55.4
1046R	CGA CAR CCA TGC ASC ACC T	53.2-55.4
343F	TAC GGR AGG CAG CAG	44.7-47.4
8F 616v	AGA GTT TGA TYM TGG CTC AG	47.7-51.8
968F	AAC GCG AAG AAC CTT AC	44.6
Bac1046Rc	CGA CGG CCA TGC ANC ACC T	55.4-57.6
GM3F	AGA GTT TGA TCM TGG C	40.8-43.4
U529R	ACC GCG GCK GCT GGC	52.9-55.6
338	TGC TGC CTC CCG TAG GAG T	55.4
1391R	GAC GGG CGG TGT GTR CA	51.9-54.3
1401R	CGG TGT GTA CAA GAC CC	49.5
1492R	TAC GGY TAC CTT GTT ACG ACT T	51.1-53
1492Rc	TAC GGT TAC CTT GTT ACG AC	49.7
27F	AGR GTT YGA TYM TGG CTC AG	47.7-55.9
347F	GGA GGC AGC AGT RRG GAA T	51.1-55.4
517F	GCC AGC AGC CGC GGT AA	54.3
518F	CCA GCA GCC GCG GTA AT	51.9
518R	ATT ACC GCG GCT GCT GG	51.9
533F	TGC CAG CAG CCG CGG TAA	54.9
533R	TTA CCG CGG CTG CTG GCA C	57.6
534R	ATT ACC GCG GCT GCT GGC	54.9
630R	CAK AAA GGA GGT GAT CC	44.6-47.1
63F	CAG GCC TAA CAC ATG CAA GTC	54.4
784F	AGG ATT AGA TAC CCT	36.5
798R	AGG GTA TCT AAT CCT	36.5
805R	GAC TAC CAG GGT ATC TAA T	46.8
8F	GGA TCC AGA CTT TGA TYM TGG CTC AG	58-61.1
8F pA	AGA GTT TGA TCC TGG CTC AG	51.8
926R	CCG TCA ATT CCT TTR AGT TT	45.6-47.7
967F	CAA CGC GAA GAA CCT TAC C	51.1

967R	GGT AAG GTT CTT CGC GTT G	51.1
Ab906F	GAA ACT TAA AKG AAT TG	35-37.4
B 1055f Bergen	ATG GCT GTC GTC AGC T	45.9
b341f	CCT ACG GGA GGC AGC AG	54.3
b785	CTA CCA GGG TAT CTA ATC C	48.9
Bac967Fb	CAA CGC GAA AAA CCT TAC C	48.9
Bact340F	TCC TAC GGG AGG CAG CAG T	55.4
Bakt 341F	CCT ACG GGN GGC WGC AG	54.3-56.7
bio-pBR5apos.SEF	GAA GAG TTT GAT CAT GGC TCA G	53
bio-pJBS-V3.SEF	GCA ACG CGA AGA ACC TTA CC	53.8
bio-pJBS-V3.SER	GGT AAG GTT CTT CGC GTT GC	53.8
B-V3.ASR	ACG ACA GCC ATG CAG CAC CT	55.9
E806R	GGA CTA CCA GGG TAT CTA AT	49.7
GM4R	TAC CTT GTT ACG ACT T	38.3
P338F	ACT CCT ACG GGA GGC AGC AG	57.9
P518R	ATT ACC GCG GCT GCT GG	51.9
P63F	CAG GCC TAA CAC ATG CAA GTC	54.4
P699D	YAA CGA GCG MRA CCC	41.9-50.1
P699R	GGG TYK CGC TCG TTR	41.9-50.1
pBR-V1.ASF	AGT GGC GGA CGG GTG AGT AA	55.9
pBR-V1.ASR	TTA CTC ACC CGT CCG CCA CT	55.9
pH	AAG GAG GTG ATC CAG CCG CA	55.9
Primer 2	ATT ACC GCG GCT GCT GG	51.9
Primer 3	GCC TAC GGG AGG CAG CAG	57.2
U1510R	GGT TAC CTT GTT ACG ACT T	46.8
U515F	GTG CCA GCM GCC GCG GTA A	57.6-59.7
U906R	CAA TTC MTT TAA GTT TC	35-37.4
UA1406R	ACG GGC GGT GTG TRC AA	49.5-51.9
Uni1390R	GAC GGG CGG TGT GTA CAA	52.6

Supplementary table 1. Primer sequences and their melting temperatures used in this study
